# Crystal structure and Hirshfeld surface analysis of a new mononuclear copper(II) complex: [bis­(pyridin-2-yl-κ*N*)amine](formato-κ*O*)(*m*-hy­droxy­benzoato-κ^2^ *O*,*O*′)copper(II)

**DOI:** 10.1107/S2056989023009234

**Published:** 2023-11-02

**Authors:** Wanassanan Chaisuriya, Kittipong Chainok, Nanthawat Wannarit

**Affiliations:** a Department of Chemistry, Faculty of Science and Technology, Thammasat University, Pathum Thani, 12120, Thailand; b Thammasat University Research Unit in Multifunctional Crystalline Materials and Applications (TU-MCMA), Faculty of Science and Technology, Thammasat University, Pathum Thani 12120, Thailand; Vienna University of Technology, Austria

**Keywords:** copper(II), ternary complex, *m*-hy­droxy­benzoate, 2,2′-di­pyridyl­amine, crystal structure, Hirshfeld surface analysis

## Abstract

A new mononuclear copper(II) complex, [Cu(C_7_H_5_O_3_)(HCO_2_)(C_10_H_9_N_3_)], containing mixed *N*- and *O*-donor ligands, 2,2′-di­pyridyl­amine (dpyam) and *m*-hy­droxy­benzoate (*m*-OHbenz), has been obtained from a solvent mixture. The coordination environment of the Cu^II^ ion is distorted square-pyramidal with a [N_2_O_3_] coordination set originating from the chelating dpyam and *m*-OHbenz ligands in the basal plane and the O atom of a formato ligand at the apical position. The crystal structure of the title complex is stabilized by N—H⋯O, O—H⋯O, C—H⋯O hydrogen-bonding, *π–π* and C—H⋯*π* inter­molecular inter­actions, which were qu­anti­fied by Hirshfeld surface analysis.

## Abstract 2

The mol­ecular structure of the ternary mononuclear copper(II) title complex shows a slightly distorted square-pyramidal coordination environment of the Cu^II^ atom with an [N_2_O_3_] coordination set.

## 1. Chemical context

Mononuclear copper(II) complexes have received great attention in several fields due to their versatile properties including anti­tumor, anti­oxidant, anti­bacterial, DNA inter­action, DNA cleavage (Huang *et al.*, 2015 ▸; Venkateswarlu *et al.*, 2022 ▸), anti­cancer (Kacar *et al.*, 2020 ▸), biological (Kumar *et al.*, 2019 ▸), industrial catalytic oxidation processes (Samanta *et al.*, 2013 ▸; Silva & Martins, 2020 ▸), magnetism (Boča *et al.*, 2017 ▸) and catalysis (Fukuzumi *et al.*, 2010 ▸).

In this context and in the scope of our research activities, we started to search for new mononuclear copper(II) complexes containing mixed *N-* and *O-*donor ligands such as bi­pyridine and benzoate derivatives and to study their catalytic properties in some organic reactions. This includes, for example, olefin epoxidation (Das *et al.*, 1997 ▸), aerobic oxidation of alcohols (Nairn *et al.*, 2006 ▸; Alaji *et al.*, 2014 ▸), ring-opening reactions (John *et al.*, 2007 ▸) and the photocatalytic oxidation of benzyl alcohol (Ranjan *et al.*, 2022 ▸). In general, the Cu^II^ ion has the [Ar]3*d* ^9^ electron configuration with an unpaired electron that can induce inter­esting magnetic properties. Copper(II) compounds also exhibit a variety of coordination environments with coordination numbers ranging from 4 to 6 (Santini *et al.*, 2014 ▸).

With this in mind, we have designed new ternary mononuclear copper(II) complexes constructed from mixed 2,2′-di­pyridyl­amine (dpyam) derivatives as *N*-donor ligands and hy­droxy­benzoate (OHbenz) derivatives as *O*-donor ligands. The dpyam ligand contains two aromatic pyridine rings that can bind in a chelating coordination mode, and together with the secondary amine (–NH–) group, supra­molecular inter­actions such as *π–π* stacking and hydrogen-bonding inter­actions are present in corresponding coordination compounds (Phiokliang *et al.*, 2019 ▸). On the other hand, OHbenz derivatives are inter­esting because of their carboxyl­ate functional group, which can exhibit a variety of coordination modes, resulting in different structural arrangements (Ziyaev *et al.*, 2021 ▸). Likewise, the presence of a hy­droxy group on the phenyl ring supports crystal stability by hydrogen-bonding inter­actions, and the different arrangement of this group (*ortho*-, *meta*-, *para*-positions) can be used to influence the crystal packing.

In order to determine crystal structures of additional members of this family of complexes, we have investigated a new mononuclear copper(II) complex with dpyam and *m*-OHbenz ligands and an additional formato ligand, [Cu(dpyam)(*m*-OHbenz)(HCO_2_)] (**I**). We report here the mol­ecular and crystal structure, spectroscopic characterizations, Hirshfeld surface analysis and 2D-fingerprint plots of this compound. 
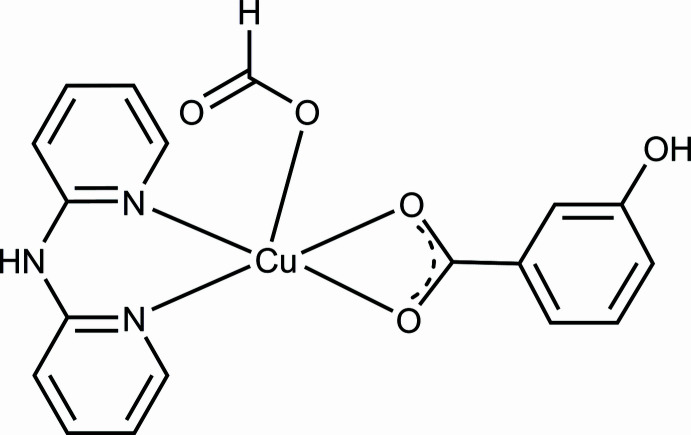


## 2. Structural commentary

**Figure 1:**
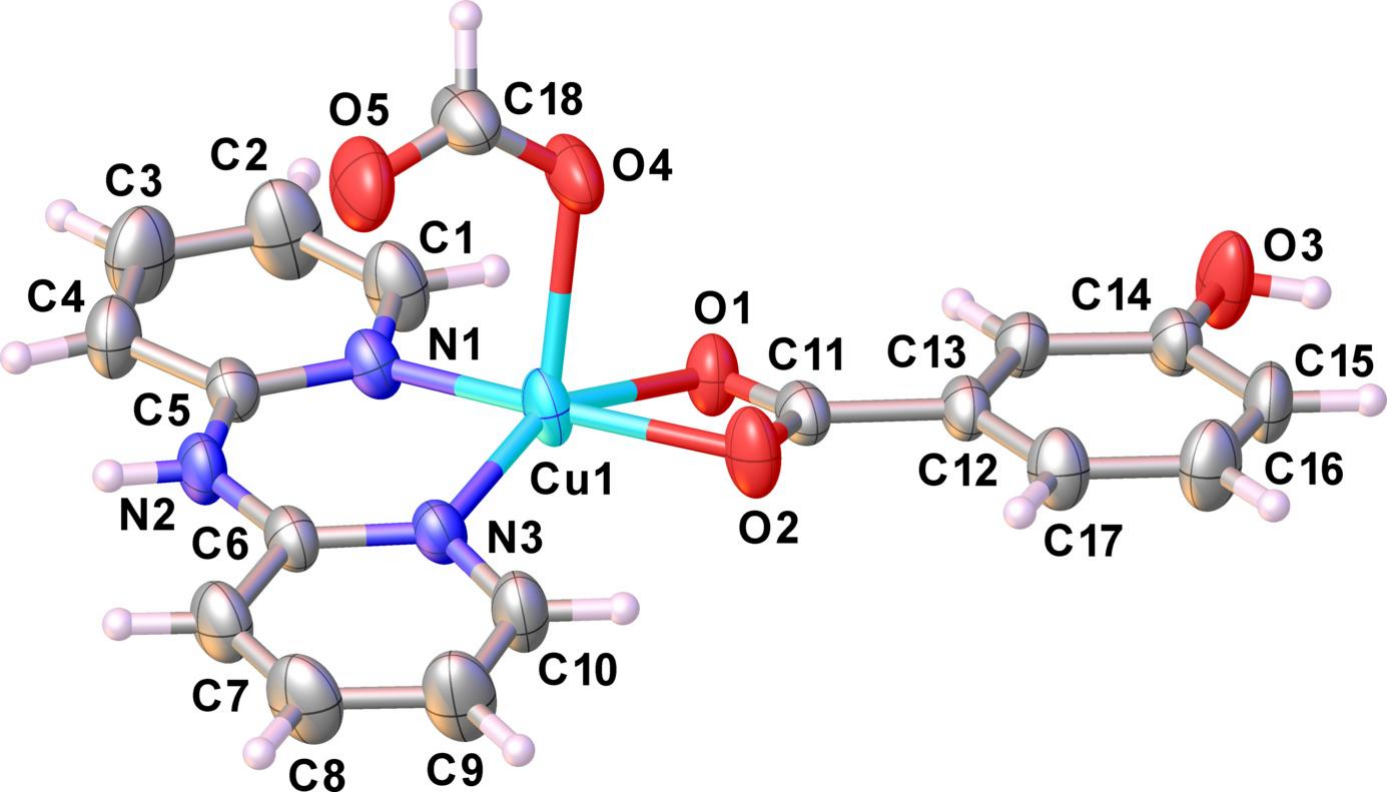
The mol­ecular structure of (**I**). Displacement ellipsoids are drawn at the 50% probability level.

Crystals of (**I**) were obtained from the reaction of Cu(NO_3_)_2_·3H_2_O, dpyam and *m*-OHbenz in mixed solvents, H_2_O/DMF ratio of 5:2. According to the synthetic conditions, the presence of the formate anion can be explained by hydrolysis of DMF (Huang *et al.*, 2012 ▸). The asymmetric unit of (**I**) consists of a Cu^II^ ion, one chelating dpyam ligand, one chelating *m*-OHbenz ligand and one monodentately binding formato ligand, as shown in Fig. 1 ▸. The Cu^II^ ion is surrounded by two pyridine nitrogen atoms of the chelating dpyam ligand, two carboxyl­ate oxygen atoms of the chelating *m*-OHbenz ligand and one oxygen atom of the formato ligand, resulting in a square-pyramidal [N_2_O_3_] coordination set. The Cu1—(N, O) bond lengths of the basal atoms originating from the chelating ligands are in the range 1.936 (3) to 2.096 (2) Å, while the Cu1—O4 bond length to the apical formato ligand is 2.207 (2) Å. Selected bond lengths and angles are summarized in Table 1 ▸. The Cu^II^ atom lies 0.265 Å above the basal plane and is oriented towards the apical oxygen atom of the formato ligand (Fig. S1*a* in the supporting information, ESI). The structural parameter τ_5_ (Addison *et al.*, 1984 ▸; Brophy *et al.*, 1999 ▸) is 0.19 and indicates a distortion of the square-pyramidal coordination (τ = 0 for an ideal square pyramid and τ = 1 for an ideal trigonal bipyramid; Fig. S1*b* in the ESI). The mol­ecular structure of (**I**) is stabilized by non-classical intra­molecular hydrogen-bonding inter­actions between C—H groups of pyridine rings and the oxygen atoms of the carboxyl­ate group of *m*-OHbenz, C1—H1⋯O1 and C10–H10⋯O2, as detailed in Table 2 ▸.

## 3. Supra­molecular features

**Figure 2:**
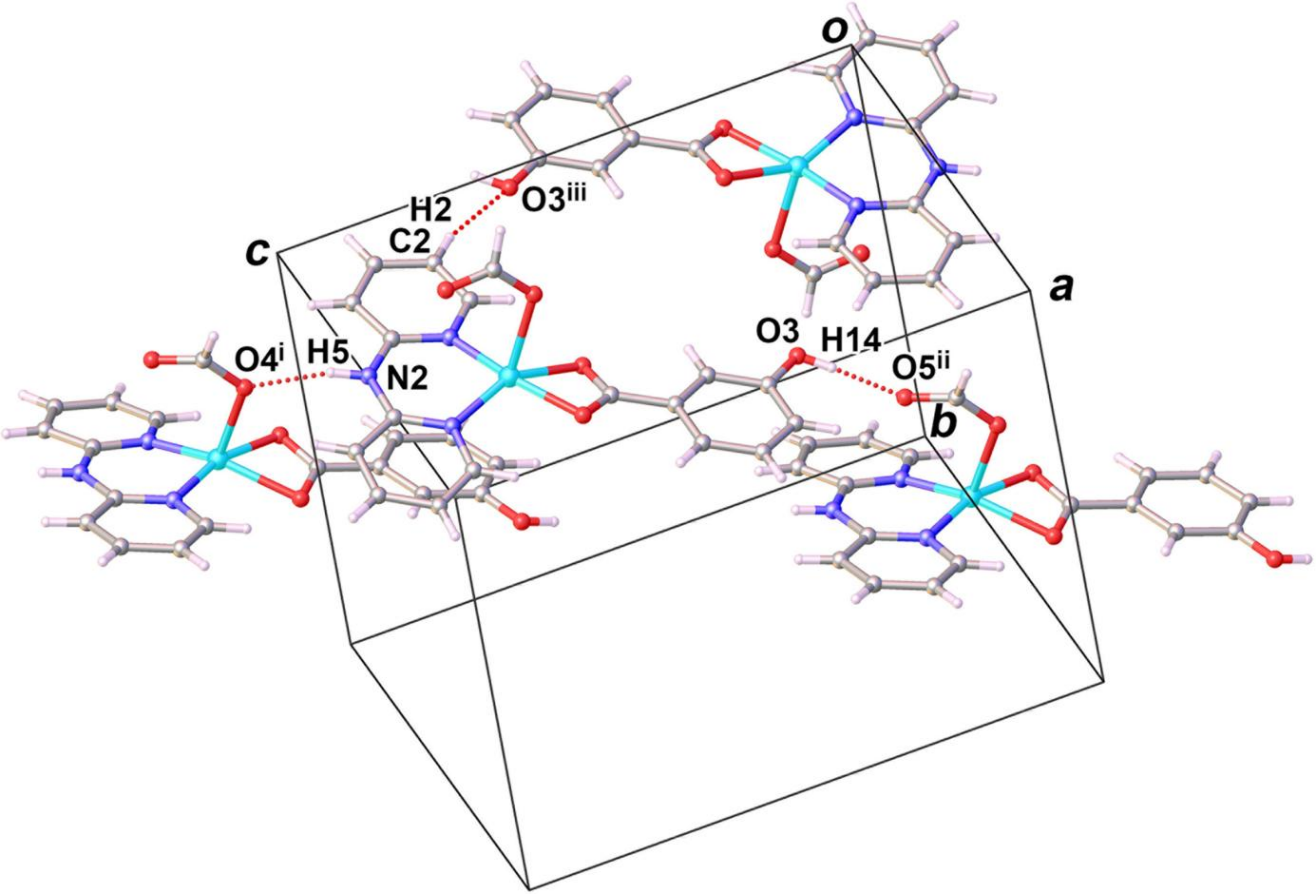
View of the hydrogen bonding inter­actions (dashed lines) in the crystal structure of (**I**). [Symmetry codes: (i) *x*, 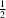 − *y*, 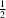 + *z*; (ii) 1 + *x*, 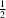 − *y*, − 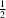 + *z*; (iii) 1 − *x*, −*y*, 1 − *z*.]

Numerical values of supra­molecular inter­actions in the crystal structure of (**I**) are collated in Table 2 ▸ and graphically displayed in Figs. 2 ▸ and 3 ▸. The crystal structure of the title complex is stabilized by the presence of inter­molecular inter­actions such as hydrogen bonding, *π*–*π* stacking and C–H⋯*π* inter­actions.

**Figure 4:**
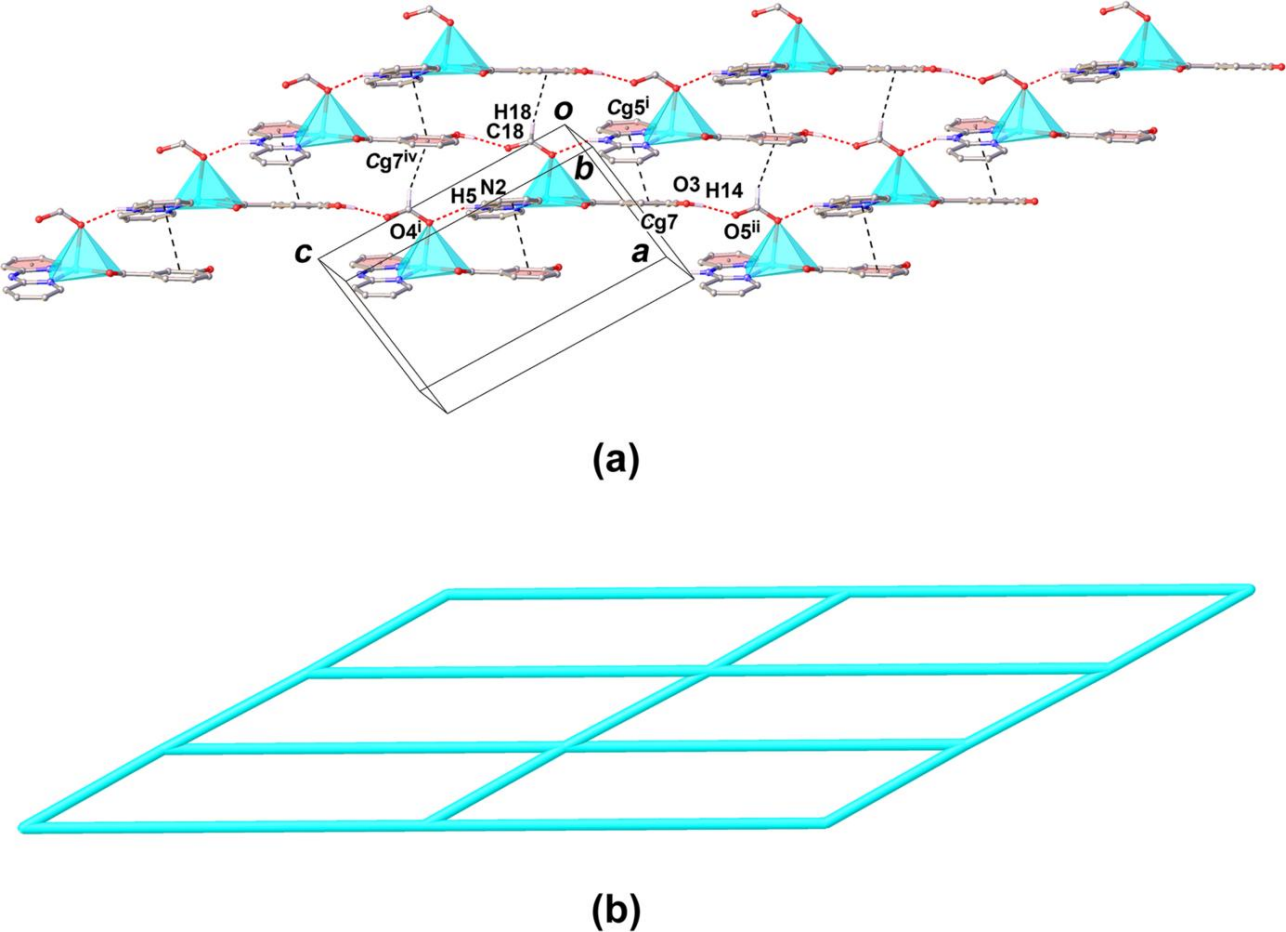
View of the layered supra­molecular network in the crystal structure of (**I**), showing (*a*) the crystal packing in the *ac* plane and (*b*) the schematic skeleton representing the Cu^II^ atoms as nodes.

**Figure 5:**
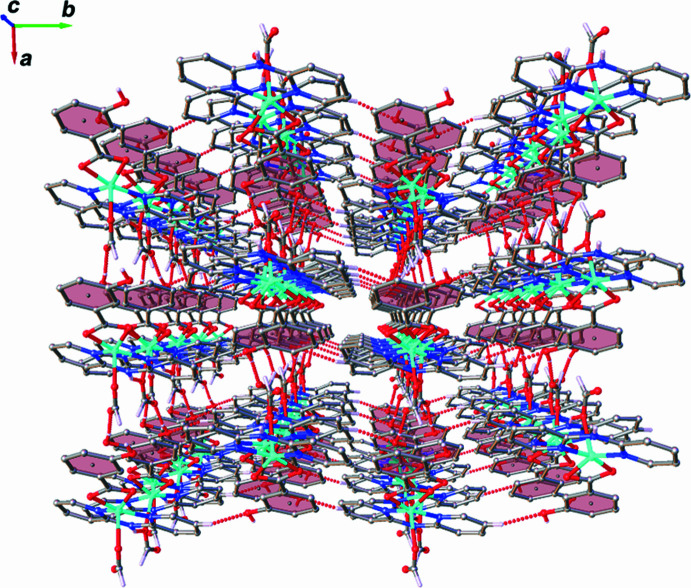
A perspective view of tri-periodic supra­molecular network of the title complex.

Classical inter­molecular hydrogen-bonding inter­actions are realized between the N—H group of dpyam and the ligating O atom of the formato carboxyl­ate group, N2—H5⋯O4^i^ (symmetry codes refer to Table 2 ▸), and between the hy­droxy group of *m*-OHbenz and the non-ligating O atom of the formato of carboxyl­ate group, O3—H14⋯O5^ii^. There is also a C—H⋯*π* inter­action between the C—H group of the formato ligand and the phenyl ring of *m*-OHbenz, C18—O18⋯*Cg*7^iv^. Notable *π*–*π* stacking inter­actions are found between one of the pyridyl rings of the dpyam ligand and the phenyl ring of the *m*-OHbenz ligand with a centroid-to-centroid distance *Cg*7⋯*C*g5^i^ of 3.978 (2) Å and a slippage of 1.431 Å (*Cg*5 and *Cg*7 are the centroids of the N1/C1–C5 and C12–C17 rings, respectively). These inter­molecular hydrogen-bonding, C—H⋯*π* and *π–π* stacking inter­actions result in supra­molecular layers extending parallel to the *ac* plane (Fig. 4 ▸). Cohesion between these layers along the *b* axis is achieved through non-classical hydrogen-bonding inter­actions between the C—H group of dpyam and the hy­droxy group of *m*-OHbenz, C2—H2⋯O3^iii^, leading to a tri-periodic supra­molecular network (Fig. 5 ▸).

## 4. Hirshfeld surface analysis

**Figure 6:**
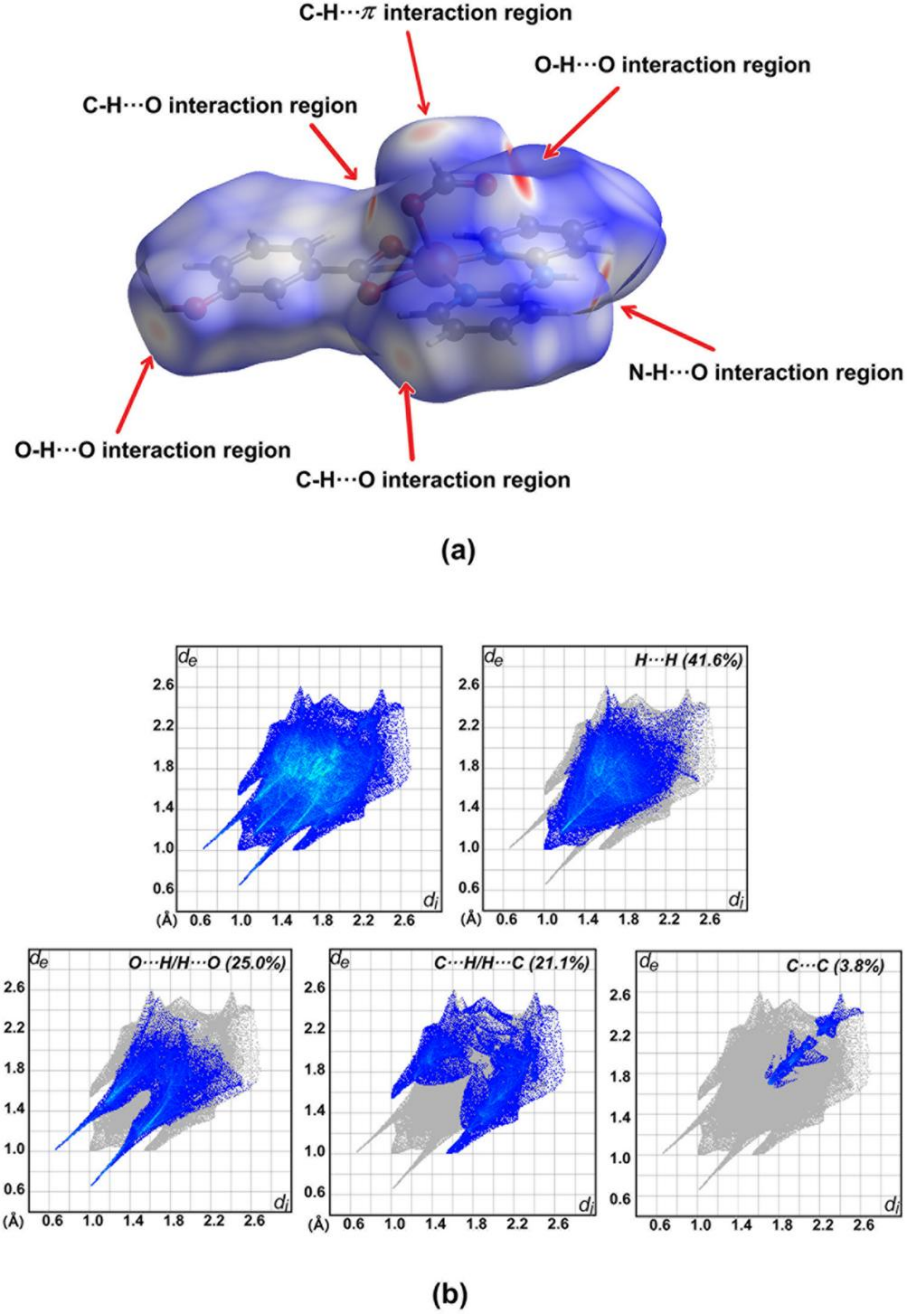
Views of (*a*) three-dimensional Hirshfeld surface mapped over *d*_norm_ and (*b*) two-dimensional fingerprint plots for the H⋯H, O⋯H/H⋯O, C⋯H/H⋯C and C⋯C contacts of the title complex.

Inter­molecular inter­actions in the crystal structure of (**I**) were qu­anti­fied by Hirshfeld surface analysis (McKinnon *et al.*, 2007 ▸) and two-dimensional fingerprint plots (Spackman & McKinnon, 2002 ▸), as shown in Fig. 6 ▸. For this purpose, *CrystalExplorer* (Spackman *et al.*, 2021 ▸) was used.

The different colors of the Hirshfeld surface mapped over *d*_norm_ relate to different distances. A red surface indicates distances shorter than the sum of the van der Waals radii, a white surface indicates distances near the sum of van der Waals radii, and a blue surface indicates distances longer than the sum of the van der Waals radii. Fig. 6 ▸ *a* displays bright-red spots on *d*_norm_ caused by hydrogen-bonding inter­actions between the N—H group of the dpyam ligand and the oxygen atom of a carboxyl­ate group, and between the hy­droxy group of the *m*-OHbenz ligand and an O atom of the formato ligand. C—H⋯O and also C—H⋯*π* inter­actions are likely represented by weaker red spots of the Hirshfeld surface. The two-dimensional fingerprint plots in Fig. 6 ▸ *b* are displayed with the corresponding percentage contribution for H⋯H, O⋯H/H⋯O, C⋯H/H⋯C and C⋯C contacts in (**I**). The H⋯H inter­molecular contacts have the highest percentage contribution of 41.6%. The O⋯H/H⋯O inter­actions contribute 25.0% to the surface. C⋯H/H⋯C contacts have a slightly lesser contribution of 21.1% and correspond mostly to C—H⋯*π* inter­actions. C⋯C contacts with a percentage contribution of 3.8% indicate *π–π* inter­actions in the crystal structure (Fig. S2 in the ESI).

## 5. Spectroscopic characterization and powder X-ray diffraction

The FT–IR spectrum of the title complex shows a characteristic broad band at 3145 cm^−1^, which is assigned to the O—H stretching vibration of the hy­droxy group of the *m*-OHbenz ligand (Zhu *et al.*, 2016 ▸). The dpyam ligand shows a band at 3204 cm^−1^ due to the N—H stretching of the secondary amine. The strong band in the region 1590 cm^−1^ results from the C=N aromatic stretching of the dpyam ligand (Chattopadhyay & Sinha, 1997 ▸). The C=O band of the chelating *m*-OHbenz ligand is present at 1648 cm^−1^ and at a higher wavenumber than the C=C aromatic vibration at 1590 cm^−1^ (Gusrizal *et al.*, 2017 ▸). The bands at 826, 768 and 686 cm^−1^ are assigned to the out-of-plane C—H bending of the *m*-OHbenz ligand (Zhu *et al.*, 2016 ▸). The COO^−^ stretching band confirms a monodentately binding metal formate species, consisting of a strong anti­symmetric COO^−^ stretching vibration at 1648 cm^−1^ and a COO^−^ symmetrical stretching at 1305 cm^−1^ (Darensbourg *et al.*, 1981 ▸). The bands at 532 and 424 cm^−1^ are assigned to Cu—N and Cu—O stretching vibrations (Saini *et al.*, 2015 ▸), as shown in Fig. S3 in the ESI).

The solid-state diffuse reflectance spectrum of the title complex (Fig. S4 in the ESI) presents two broad peaks with *λ*_max_ at 425 and 645 nm. This feature is assigned to the electronic *d*–*d* transitions of 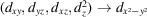 corres­ponding to the square-pyramidal coordination environment of Cu^II^ (Kucková *et al.*, 2015 ▸).

The powder X-ray diffraction pattern of the title complex (Fig. S5 in the ESI) shows a close match between the experimental data and the simulated pattern, confirming a single-phase material.

## 6. Database survey

A search of the Cambridge Structural Database (CSD, version 5.42, September 2021 update; Bruno *et al.*, 2002 ▸; Groom *et al.*, 2016 ▸) for structures of ternary mononuclear Cu^II^ complexes containing dpyam and hy­droxy­benzoate derivatives, resulted in two closely related complexes, [Cu(dpyam)(*p*-OHbenz)Cl] (where *p*-OHbenz represents *p*-hy­droxy­benzoate; PASCIW, Wang *et al.*, 2005 ▸) and [Cu(dpyam)(benz)Cl] (where benz represents benzoate; YIDQEI, Okabe *et al.*, 2007 ▸). Both complexes likewise exhibit a square-pyramidal coordination environment with *τ*_5_ values of 0.03 and 0.00, respectively. In comparison with (**I**), the lower *τ*_5_ values can be attributed to the diminished steric impact resulting from the presence of benzoate and *p*-OHbenz moieties.

## 7. Synthesis and crystallization

Cu(NO_3_)_2_·3H_2_O (0.2416 g, 1 mmol) was dissolved in distilled water (10 ml), and the blue solution was heated at 338 K and stirred. Then, a solution of dpyam (0.1712 g, 1 mmol) in DMF (5 ml) was added, resulting in a clear green solution. Subsequently, a mixed solution of *m*-hy­droxy­benzoic acid (0.2762 g, 2 mmol) and sodium hydroxide (0.0866 g, 2 mmol) in distilled water (5 ml) was slowly added, resulting in a dark green solution. A mixed solution of distilled water and DMF (1:1 v:v, 10 ml) was added and continuously stirred for 30 min. Then, the reaction mixture was filtrated and allowed to stand and slowly evaporate in air at room temperature for 2 d. Green block-like crystals of the title copper(II) complex were obtained with a yield of 10.1% [based on the copper(II) salt].

## 8. Refinement

Crystal data, data collection and structure refinement details are summarized in Table 3 ▸. All hydrogen atoms were placed in geometrically calculated positions and refined with a riding model, with C—H = 0.93 Å and *U*_iso_(H) = 1.2*U*_eq_(C), N—H = 0.86 Å and *U*_iso_(H) = 1.2*U*_eq_(N) and O—H = 0.82 Å and *U*_iso_(H) = 1.5*U*_eq_(O).

## Supplementary Material

Crystal structure: contains datablock(s) I. DOI: 10.1107/S2056989023009234/wm5700sup1.cif

Structure factors: contains datablock(s) I. DOI: 10.1107/S2056989023009234/wm5700Isup2.hkl

Supporting information file consists of additional structural data, spectra and pxrd patterns. DOI: 10.1107/S2056989023009234/wm5700sup3.pdf

CCDC reference: 2302393

Additional supporting information:  crystallographic information; 3D view; checkCIF report

## Figures and Tables

**Figure 3:**
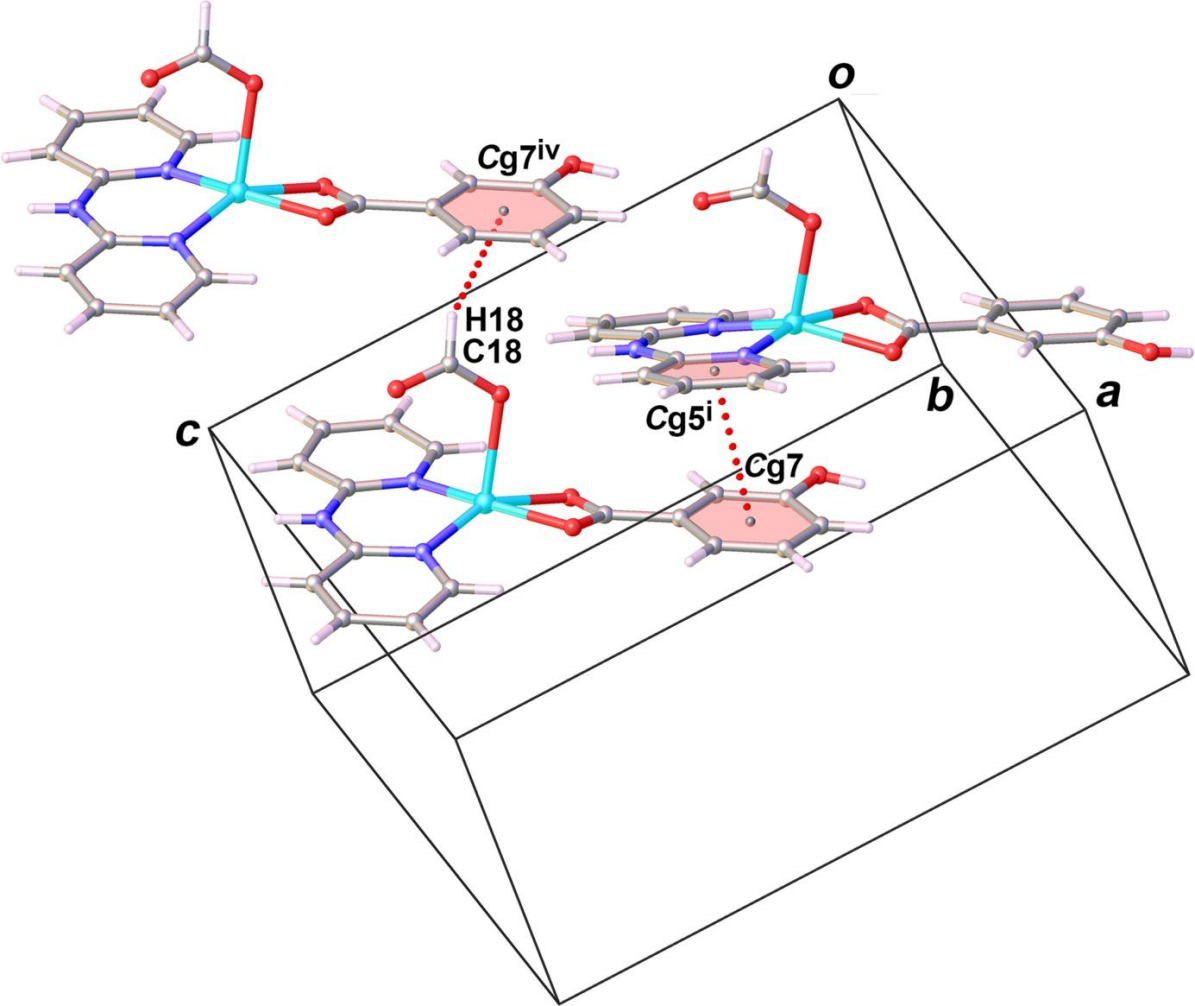
View of *π*–*π* stacking and C—H⋯*π* inter­actions (dashed lines) in the crystal structure of (**I**) [Symmetry code: (i) *x*, 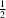 − *y*, −1/2 + *z;* (iv) −1 + *x*, *y*, *z*; *Cg*5 and *C*g7 are the centroids of the N1/C1–C5 and C12–C17 rings, respectively.]

**Table 1:** Selected geometric parameters (Å, °)

Cu1—O1	2.096 (2)	Cu1—N3	1.963 (2)
Cu1—O2	1.984 (2)	Cu1—N1	1.936 (3)
Cu1—O4	2.207 (2)		
			
O1—Cu1—O4	89.16 (9)	N3—Cu1—O4	114.49 (10)
O2—Cu1—O1	64.40 (9)	N1—Cu1—O1	99.43 (9)
O2—Cu1—O4	90.64 (10)	N1—Cu1—O2	163.39 (10)
N3—Cu1—O1	151.93 (10)	N1—Cu1—O4	92.99 (10)
N3—Cu1—O2	98.82 (10)	N1—Cu1—N3	94.41 (10)

**Table 2:** Hydrogen-bond geometry (Å, °)

*D*—H⋯*A*	*D*—H	H⋯*A*	*D*⋯*A*	*D*—H⋯*A*
C1—H1⋯O1	0.93	2.42	3.056 (4)	125
C10—H10⋯O2	0.93	2.38	2.999 (4)	124
N2—H5⋯O4^i^	0.86	2.02	2.834 (3)	158
O3—H14⋯O5^ii^	0.82	1.83	2.645 (4)	170
C2—H2⋯O3^iii^	0.93	2.59	3.497 (5)	166
C18—H18⋯*C*g7^iv^	0.93	2.88	3.634 (3)	139

Symmetry codes: (i) 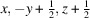 ; (ii) 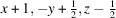 ; (iii) 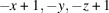 ; (iv) 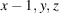 .

**Table 3:** Experimental details

Crystal data	
Chemical formula	[Cu(C_7_H_5_O_3_)(HCO_2_)(C_10_H_9_N_3_)]
*M*_r_	416.87
Crystal system, space group	Monoclinic, *P*2_1_/*c*
Temperature (K)	296
*a*, *b*, *c* (Å)	8.3265 (3), 14.9699 (5), 13.9523 (4)
β (°)	99.242 (1)
*V* (Å^3^)	1716.53 (10)
*Z*	4
Radiation type	Mo *K*α
μ (mm^−1^)	1.31
Crystal size (mm)	0.12 × 0.10 × 0.10

Data collection	
Diffractometer	Bruker D8 Quest Cmos Photon-II
Absorption correction	Multi-scan (*SADABS*; Krause *et al.*, 2015 ▸)
*T*_min_, *T*_max_	0.650, 0.746
No. of measured, independent and observed [*I* > 2σ(*I*)] reflections	18907, 4262, 2779
*R*_int_	0.075
(sin θ/λ)_max_ (Å^−1^)	0.667

Refinement	
*R*[*F* ^2^ > 2σ(*F* ^2^)], *wR*(*F* ^2^), *S*	0.046, 0.115, 1.01
No. of reflections	4262
No. of parameters	246
H-atom treatment	H-atom parameters constrained
Δρ_max_, Δρ_min_ (e Å^−3^)	0.38, −0.54

Computer programs: *APEX3* and *SAINT* (Bruker, 2016 ▸), *SHELXT* (Sheldrick, 2015*a* ▸), *SHELXL* (Sheldrick, 2015*b* ▸), and *OLEX2* (Dolomanov *et al.*, 2009 ▸), *publCIF* (Westrip, 2010 ▸).

## supplementary crystallographic information

### Crystal data

**Table:**

[Cu(C_7_H_5_O_3_)(HCO_2_)(C_10_H_9_N_3_)]	*F*(000) = 852
*M**_r_* = 416.87	*D*_x_ = 1.613 Mg m^−^^3^
Monoclinic, *P*2_1_/*c*	Mo *K*α radiation, λ = 0.71073 Å
*a* = 8.3265 (3) Å	Cell parameters from 2887 reflections
*b* = 14.9699 (5) Å	θ = 2.8–27.7°
*c* = 13.9523 (4) Å	µ = 1.31 mm^−^^1^
β = 99.242 (1)°	*T* = 296 K
*V* = 1716.53 (10) Å^3^	Block, clear dark green
*Z* = 4	0.12 × 0.10 × 0.10 mm

### Data collection

**Table:**

Bruker D8 Quest Cmos Photon-II diffractometer	4262 independent reflections
Radiation source: sealed x-ray tube, Mo	2779 reflections with *I* > 2σ(*I*)
Graphite monochromator	*R*_int_ = 0.075
Detector resolution: 7.39 pixels mm^-1^	θ_max_ = 28.3°, θ_min_ = 3.0°
φ and ω scans	*h* = −10→11
Absorption correction: multi-scan (SADABS; Krause *et al.*, 2015)	*k* = −19→19
*T*_min_ = 0.650, *T*_max_ = 0.746	*l* = −18→17
18907 measured reflections	

### Refinement

**Table:**

Refinement on *F*^2^	Hydrogen site location: inferred from neighbouring sites
Least-squares matrix: full	H-atom parameters constrained
*R*[*F*^2^ > 2σ(*F*^2^)] = 0.046	*w* = 1/[σ^2^(*F*_o_^2^) + (0.0412*P*)^2^ + 1.0329*P*] where *P* = (*F*_o_^2^ + 2*F*_c_^2^)/3
*wR*(*F*^2^) = 0.115	(Δ/σ)_max_ = 0.001
*S* = 1.01	Δρ_max_ = 0.38 e Å^−^^3^
4262 reflections	Δρ_min_ = −0.54 e Å^−^^3^
246 parameters	Extinction correction: *SHELXL2016/6* (Sheldrick 2015b), Fc^*^=kFc[1+0.001xFc^2^λ^3^/sin(2θ)]^-1/4^
0 restraints	Extinction coefficient: 0.0021 (7)
Primary atom site location: dual	

### Special details

**Table:**

Geometry. All esds (except the esd in the dihedral angle between two l.s. planes) are estimated using the full covariance matrix. The cell esds are taken into account individually in the estimation of esds in distances, angles and torsion angles; correlations between esds in cell parameters are only used when they are defined by crystal symmetry. An approximate (isotropic) treatment of cell esds is used for estimating esds involving l.s. planes.

### Fractional atomic coordinates and isotropic or equivalent isotropic displacement parameters (Å^2^)

**Table:**

	*x*	*y*	*z*	*U*_iso_*/*U*_eq_	
Cu1	0.29575 (5)	0.27751 (3)	0.72313 (2)	0.03822 (15)	
O1	0.4328 (3)	0.21333 (15)	0.62997 (15)	0.0414 (5)	
O2	0.3988 (3)	0.35716 (15)	0.63693 (16)	0.0472 (6)	
O4	0.0779 (3)	0.26399 (18)	0.61092 (15)	0.0518 (7)	
N3	0.2728 (3)	0.36345 (17)	0.82630 (16)	0.0339 (6)	
O3	0.7085 (3)	0.17132 (17)	0.33624 (19)	0.0596 (7)	
H14	0.766121	0.186084	0.296455	0.089*	
N1	0.2382 (3)	0.17503 (18)	0.79522 (16)	0.0358 (6)	
O5	−0.0791 (3)	0.2937 (2)	0.71924 (18)	0.0625 (8)	
N2	0.1539 (3)	0.25934 (17)	0.92169 (17)	0.0375 (6)	
H5	0.104666	0.255311	0.971172	0.045*	
C12	0.5440 (3)	0.3037 (2)	0.5146 (2)	0.0322 (7)	
C11	0.4562 (4)	0.2903 (2)	0.5980 (2)	0.0340 (7)	
C6	0.2023 (4)	0.3445 (2)	0.90355 (19)	0.0339 (7)	
C14	0.6699 (4)	0.2441 (2)	0.3845 (2)	0.0372 (7)	
C5	0.1693 (4)	0.1795 (2)	0.8759 (2)	0.0335 (7)	
C13	0.5860 (4)	0.2308 (2)	0.4624 (2)	0.0340 (7)	
H13	0.558288	0.173368	0.479089	0.041*	
C10	0.3265 (4)	0.4479 (2)	0.8168 (2)	0.0471 (9)	
H10	0.377537	0.460746	0.763805	0.057*	
C1	0.2504 (5)	0.0941 (2)	0.7541 (2)	0.0507 (9)	
H1	0.301024	0.090414	0.699464	0.061*	
C18	−0.0541 (4)	0.2676 (3)	0.6408 (2)	0.0484 (9)	
H18	−0.144591	0.248072	0.598033	0.058*	
C7	0.1792 (4)	0.4109 (2)	0.9711 (2)	0.0475 (9)	
H7	0.128076	0.397072	1.023790	0.057*	
C15	0.7091 (4)	0.3303 (2)	0.3605 (2)	0.0454 (8)	
H15	0.763974	0.339829	0.308385	0.054*	
C17	0.5852 (4)	0.3886 (2)	0.4908 (2)	0.0433 (8)	
H17	0.558114	0.437171	0.526581	0.052*	
C4	0.1112 (4)	0.1025 (2)	0.9160 (2)	0.0470 (8)	
H4	0.066687	0.105991	0.972858	0.056*	
C8	0.2316 (5)	0.4955 (3)	0.9592 (3)	0.0577 (10)	
H8	0.215443	0.539988	1.003189	0.069*	
C16	0.6674 (5)	0.4016 (2)	0.4132 (3)	0.0519 (9)	
H16	0.694665	0.459158	0.396586	0.062*	
C9	0.3100 (5)	0.5150 (2)	0.8804 (3)	0.0606 (11)	
H9	0.349664	0.571996	0.871705	0.073*	
C3	0.1206 (5)	0.0226 (3)	0.8707 (3)	0.0607 (11)	
H3	0.078774	−0.028635	0.895207	0.073*	
C2	0.1921 (5)	0.0181 (3)	0.7887 (3)	0.0640 (11)	
H2	0.200367	−0.036176	0.757471	0.077*	

### Atomic displacement parameters (Å^2^)

**Table:**

	*U*^11^	*U*^22^	*U*^33^	*U*^12^	*U*^13^	*U*^23^
Cu1	0.0561 (3)	0.0362 (2)	0.02653 (19)	0.0031 (2)	0.01924 (16)	0.00029 (17)
O1	0.0506 (14)	0.0404 (13)	0.0386 (11)	0.0023 (11)	0.0235 (10)	0.0035 (10)
O2	0.0669 (16)	0.0404 (13)	0.0419 (12)	0.0066 (12)	0.0315 (11)	−0.0015 (10)
O4	0.0438 (14)	0.0859 (19)	0.0281 (10)	0.0102 (13)	0.0132 (10)	0.0015 (12)
N3	0.0427 (15)	0.0346 (14)	0.0257 (11)	−0.0026 (11)	0.0094 (10)	−0.0015 (11)
O3	0.081 (2)	0.0488 (15)	0.0614 (16)	−0.0128 (14)	0.0506 (14)	−0.0157 (13)
N1	0.0461 (16)	0.0363 (15)	0.0262 (12)	0.0037 (12)	0.0101 (11)	0.0005 (11)
O5	0.0564 (16)	0.086 (2)	0.0521 (15)	0.0092 (14)	0.0301 (12)	0.0011 (14)
N2	0.0468 (16)	0.0416 (16)	0.0282 (12)	−0.0035 (12)	0.0181 (11)	−0.0026 (11)
C12	0.0303 (16)	0.0409 (17)	0.0277 (13)	0.0026 (13)	0.0111 (12)	0.0030 (12)
C11	0.0331 (16)	0.0431 (19)	0.0272 (13)	0.0034 (14)	0.0092 (12)	−0.0007 (13)
C6	0.0372 (17)	0.0403 (17)	0.0259 (13)	0.0034 (14)	0.0098 (12)	0.0001 (13)
C14	0.0400 (18)	0.0422 (18)	0.0325 (14)	−0.0036 (14)	0.0155 (13)	−0.0036 (13)
C5	0.0380 (17)	0.0379 (17)	0.0253 (13)	0.0005 (14)	0.0071 (12)	0.0042 (13)
C13	0.0347 (16)	0.0371 (17)	0.0327 (14)	−0.0028 (14)	0.0125 (12)	−0.0001 (13)
C10	0.065 (2)	0.045 (2)	0.0359 (16)	−0.0065 (17)	0.0215 (16)	−0.0006 (15)
C1	0.081 (3)	0.0353 (19)	0.0411 (18)	0.0041 (18)	0.0251 (17)	−0.0031 (15)
C18	0.0387 (19)	0.072 (3)	0.0345 (16)	0.0068 (18)	0.0056 (14)	−0.0033 (17)
C7	0.062 (2)	0.048 (2)	0.0361 (17)	0.0022 (18)	0.0205 (16)	−0.0055 (15)
C15	0.051 (2)	0.051 (2)	0.0393 (17)	−0.0052 (17)	0.0245 (15)	0.0055 (16)
C17	0.054 (2)	0.0374 (18)	0.0427 (17)	0.0057 (16)	0.0205 (15)	0.0007 (15)
C4	0.057 (2)	0.047 (2)	0.0418 (17)	−0.0033 (17)	0.0206 (16)	0.0082 (16)
C8	0.085 (3)	0.046 (2)	0.0462 (19)	0.003 (2)	0.0234 (19)	−0.0145 (17)
C16	0.069 (2)	0.0376 (19)	0.056 (2)	−0.0034 (18)	0.0285 (18)	0.0064 (16)
C9	0.097 (3)	0.038 (2)	0.051 (2)	−0.012 (2)	0.026 (2)	−0.0100 (17)
C3	0.083 (3)	0.040 (2)	0.065 (2)	−0.005 (2)	0.029 (2)	0.0090 (18)
C2	0.104 (3)	0.035 (2)	0.058 (2)	0.000 (2)	0.029 (2)	−0.0011 (17)

### Geometric parameters (Å, º)

**Table:**

Cu1—O1	2.096 (2)	C14—C15	1.385 (5)
Cu1—O2	1.984 (2)	C5—C4	1.400 (4)
Cu1—O4	2.207 (2)	C13—H13	0.9300
Cu1—N3	1.963 (2)	C10—H10	0.9300
Cu1—N1	1.936 (3)	C10—C9	1.363 (5)
Cu1—C11	2.370 (3)	C1—H1	0.9300
O1—C11	1.262 (4)	C1—C2	1.357 (5)
O2—C11	1.268 (4)	C18—H18	0.9300
O4—C18	1.238 (4)	C7—H7	0.9300
N3—C6	1.338 (4)	C7—C8	1.357 (5)
N3—C10	1.354 (4)	C15—H15	0.9300
O3—H14	0.8200	C15—C16	1.372 (5)
O3—C14	1.346 (4)	C17—H17	0.9300
N1—C5	1.344 (4)	C17—C16	1.385 (4)
N1—C1	1.351 (4)	C4—H4	0.9300
O5—C18	1.211 (4)	C4—C3	1.360 (5)
N2—H5	0.8600	C8—H8	0.9300
N2—C6	1.373 (4)	C8—C9	1.397 (5)
N2—C5	1.371 (4)	C16—H16	0.9300
C12—C11	1.484 (4)	C9—H9	0.9300
C12—C13	1.388 (4)	C3—H3	0.9300
C12—C17	1.371 (4)	C3—C2	1.374 (5)
C6—C7	1.405 (4)	C2—H2	0.9300
C14—C13	1.397 (4)		
			
O1—Cu1—O4	89.16 (9)	N1—C5—N2	121.2 (3)
O1—Cu1—C11	32.08 (9)	N1—C5—C4	121.0 (3)
O2—Cu1—O1	64.40 (9)	N2—C5—C4	117.9 (3)
O2—Cu1—O4	90.64 (10)	C12—C13—C14	119.8 (3)
O2—Cu1—C11	32.35 (10)	C12—C13—H13	120.1
O4—Cu1—C11	88.92 (9)	C14—C13—H13	120.1
N3—Cu1—O1	151.93 (10)	N3—C10—H10	118.2
N3—Cu1—O2	98.82 (10)	N3—C10—C9	123.7 (3)
N3—Cu1—O4	114.49 (10)	C9—C10—H10	118.2
N3—Cu1—C11	128.32 (11)	N1—C1—H1	118.5
N1—Cu1—O1	99.43 (9)	N1—C1—C2	123.0 (3)
N1—Cu1—O2	163.39 (10)	C2—C1—H1	118.5
N1—Cu1—O4	92.99 (10)	O4—C18—H18	116.2
N1—Cu1—N3	94.41 (10)	O5—C18—O4	127.6 (3)
N1—Cu1—C11	131.48 (11)	O5—C18—H18	116.2
C11—O1—Cu1	86.03 (17)	C6—C7—H7	120.1
C11—O2—Cu1	90.84 (19)	C8—C7—C6	119.8 (3)
C18—O4—Cu1	115.5 (2)	C8—C7—H7	120.1
C6—N3—Cu1	124.0 (2)	C14—C15—H15	119.8
C6—N3—C10	118.1 (3)	C16—C15—C14	120.4 (3)
C10—N3—Cu1	117.8 (2)	C16—C15—H15	119.8
C14—O3—H14	109.5	C12—C17—H17	120.2
C5—N1—Cu1	124.8 (2)	C12—C17—C16	119.6 (3)
C5—N1—C1	118.1 (3)	C16—C17—H17	120.2
C1—N1—Cu1	116.6 (2)	C5—C4—H4	120.4
C6—N2—H5	113.9	C3—C4—C5	119.3 (3)
C5—N2—H5	113.9	C3—C4—H4	120.4
C5—N2—C6	132.2 (3)	C7—C8—H8	120.3
C13—C12—C11	120.2 (3)	C7—C8—C9	119.4 (3)
C17—C12—C11	119.3 (3)	C9—C8—H8	120.3
C17—C12—C13	120.5 (3)	C15—C16—C17	120.6 (3)
O1—C11—Cu1	61.89 (15)	C15—C16—H16	119.7
O1—C11—O2	118.6 (3)	C17—C16—H16	119.7
O1—C11—C12	121.6 (3)	C10—C9—C8	117.9 (3)
O2—C11—Cu1	56.81 (15)	C10—C9—H9	121.1
O2—C11—C12	119.7 (3)	C8—C9—H9	121.1
C12—C11—Cu1	174.5 (2)	C4—C3—H3	120.2
N3—C6—N2	121.7 (3)	C4—C3—C2	119.6 (4)
N3—C6—C7	121.0 (3)	C2—C3—H3	120.2
N2—C6—C7	117.3 (3)	C1—C2—C3	118.9 (4)
O3—C14—C13	117.6 (3)	C1—C2—H2	120.6
O3—C14—C15	123.2 (3)	C3—C2—H2	120.6
C15—C14—C13	119.1 (3)		
			
Cu1—O1—C11—O2	2.9 (3)	C6—N2—C5—N1	−2.5 (5)
Cu1—O1—C11—C12	−175.1 (3)	C6—N2—C5—C4	177.4 (3)
Cu1—O2—C11—O1	−3.1 (3)	C6—C7—C8—C9	−0.8 (6)
Cu1—O2—C11—C12	175.0 (2)	C14—C15—C16—C17	−0.2 (6)
Cu1—O4—C18—O5	14.8 (6)	C5—N1—C1—C2	2.0 (5)
Cu1—N3—C6—N2	6.6 (4)	C5—N2—C6—N3	3.7 (5)
Cu1—N3—C6—C7	−175.3 (2)	C5—N2—C6—C7	−174.5 (3)
Cu1—N3—C10—C9	176.4 (3)	C5—C4—C3—C2	2.4 (6)
Cu1—N1—C5—N2	−9.0 (4)	C13—C12—C11—O1	7.7 (4)
Cu1—N1—C5—C4	171.1 (2)	C13—C12—C11—O2	−170.4 (3)
Cu1—N1—C1—C2	−170.0 (3)	C13—C12—C17—C16	0.9 (5)
N3—C6—C7—C8	−1.3 (5)	C13—C14—C15—C16	0.7 (5)
N3—C10—C9—C8	−0.7 (6)	C10—N3—C6—N2	−175.8 (3)
O3—C14—C13—C12	179.8 (3)	C10—N3—C6—C7	2.3 (5)
O3—C14—C15—C16	−179.5 (3)	C1—N1—C5—N2	179.6 (3)
N1—C5—C4—C3	−1.9 (5)	C1—N1—C5—C4	−0.2 (5)
N1—C1—C2—C3	−1.6 (6)	C7—C8—C9—C10	1.8 (6)
N2—C6—C7—C8	176.9 (3)	C15—C14—C13—C12	−0.4 (5)
N2—C5—C4—C3	178.2 (3)	C17—C12—C11—O1	−171.4 (3)
C12—C17—C16—C15	−0.5 (6)	C17—C12—C11—O2	10.6 (4)
C11—C12—C13—C14	−179.4 (3)	C17—C12—C13—C14	−0.4 (5)
C11—C12—C17—C16	179.9 (3)	C4—C3—C2—C1	−0.7 (6)
C6—N3—C10—C9	−1.3 (5)		

### Hydrogen-bond geometry (Å, º)

**Table:**

*D*—H···*A*	*D*—H	H···*A*	*D*···*A*	*D*—H···*A*
C1—H1···O1	0.93	2.42	3.056 (4)	125
C10—H10···O2	0.93	2.38	2.999 (4)	124
N2—H5···O4^i^	0.86	2.02	2.834 (3)	158
O3—H14···O5^ii^	0.82	1.83	2.645 (4)	170
C2—H2···O3^iii^	0.93	2.59	3.497 (5)	166
C18—H18···*C*g7^iv^	0.93	2.88	3.634 (3)	139

Symmetry codes: (i) *x*, −*y*+1/2, *z*+1/2; (ii) *x*+1, −*y*+1/2, *z*−1/2; (iii) −*x*+1, −*y*, −*z*+1; (iv) *x*−1, *y*, *z*.
