# Supplementary material for: Crystal structure and Hirshfeld surface analysis of a new mononuclear copper(II) complex: [bis­(pyridin-2-yl-κN)amine](formato-κO)(m-hy­droxy­benzoato-κ2 O,O′)copper(II)
Source: Acta Crystallogr E Crystallogr Commun. 2023 Nov 2;79(Pt 12):1115–20. doi: 10.1107/S2056989023009234 (PMC10833405; doi:10.1107/S2056989023009234)
Supplement: Supplementary file 3 [file e-79-01115-sup3.pdf]

Supporting Information

Crystal structure and Hirshfeld surface analysis of a new mononuclear copper(II) complex: [bis(pyridin-2-yl-*κN*)amine](formato-*κO*)(*m*-hydroxybenzoato-*κ*^{2}*O,O′*)copper(II)

Wanassanan Chaisuriya[a], Kittipong Chainok[b] and Nanthawat Wannarit[a,b*]

[a] Department of Chemistry, Faculty of Science and Technology, Thammasat University, Pathum Thani, 12120, Thailand

[b] Thammasat University Research Unit in Multifunctional Crystalline Materials and Applications (TU-MCMA), Faculty of Science and Technology, Thammasat University, Pathum Thani 12120, Thailand

List of Figures

Fig. S1 Views of (a) the Cu^{II} atom lies above the basal plane towards the apical oxygen atom of the formato ligand and (b) the τ_5 parameter of Cu^{II} center in the title complex.

Fig. S2 Quantitative results of different intermolecular contacts contributing to the Hirshfeld surface of the title complex.

Fig. S3 The FT-IR spectrum of the title complex.

Fig. S4 The solid-state diffuse reflectance spectrum of the title complex.

Fig. S5 The powder X-ray diffraction patterns of the title complex.

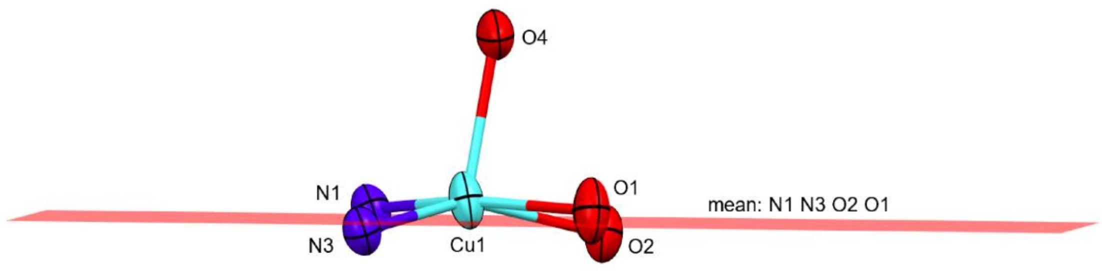

(a)

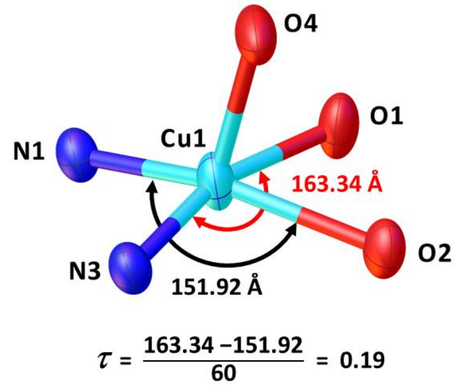

(b)

Fig. S1 Views of (a) the Cu^{II} atom lies above the basal plane towards the apical oxygen atom of the formato ligand and (b) the τ_5 parameter of Cu^{II} center in the title complex.

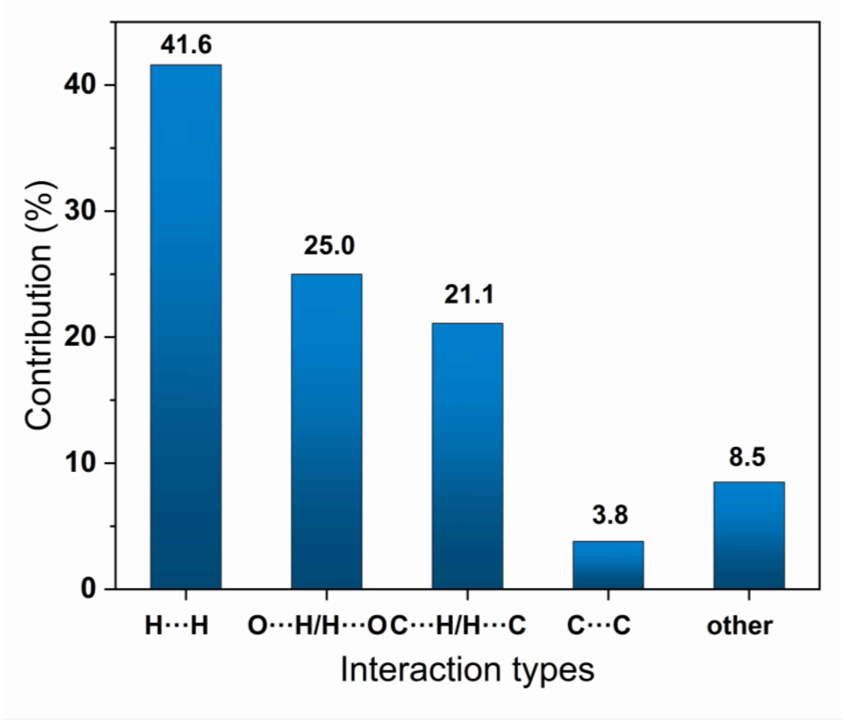

Fig. S2 Quantitative results of different intermolecular contacts contributing to the Hirshfeld surface of the title complex.

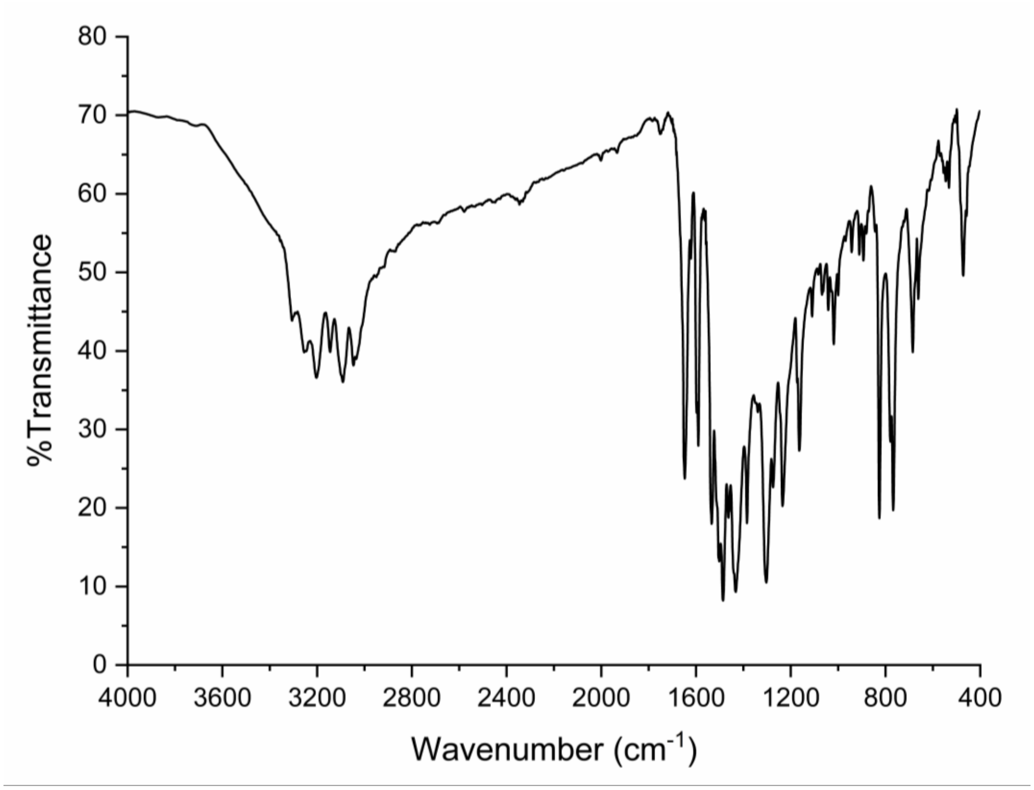

Fig. S3 The FT-IR spectrum of the title complex.

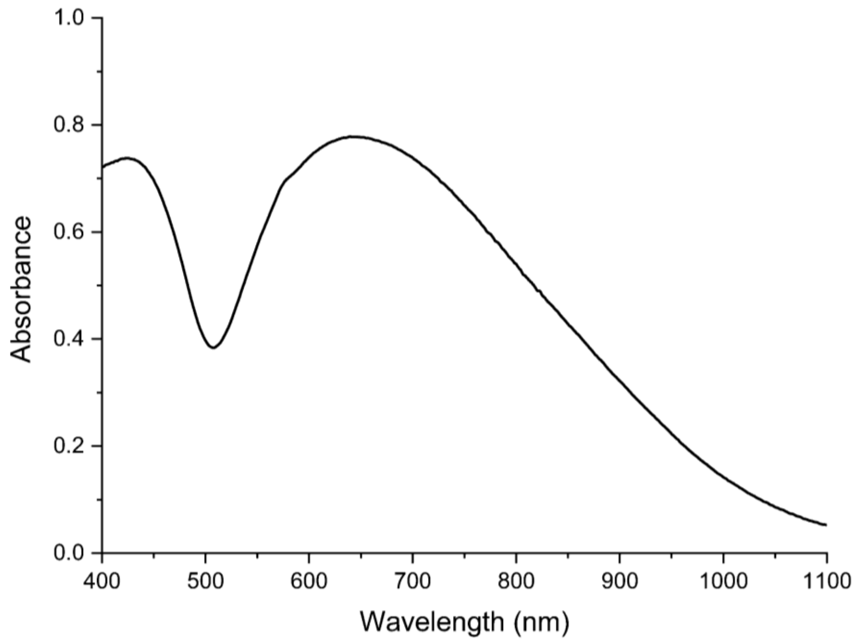

Fig. S4 The solid-state diffuse reflectance spectrum of the title complex.

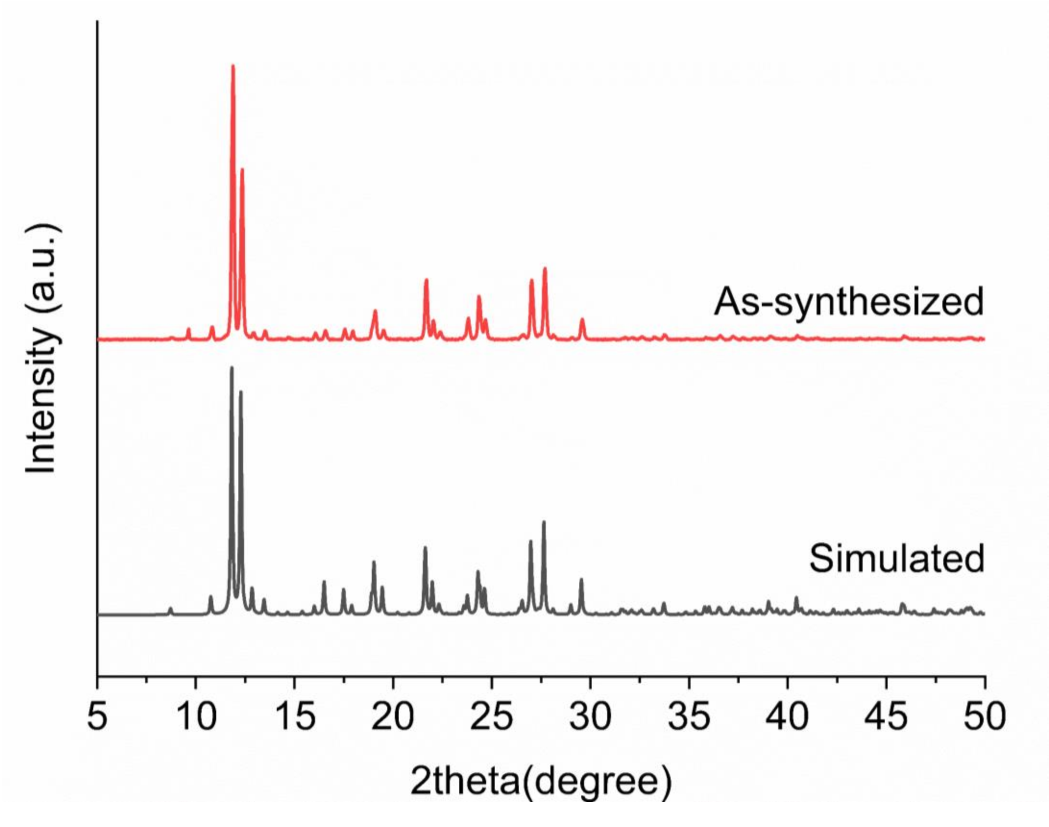

Fig. S5 The powder X-ray diffraction patterns of the title complex.
